# Confronting "Socio-Political Inertia" on the Long and Winding Road to "Healthy Societies": Comment on "How to Build Healthy Societies: A Thematic Analysis of Relevant Conceptual Frameworks"

**DOI:** 10.34172/ijhpm.9150

**Published:** 2025-05-27

**Authors:** Harvy Joy Liwanag, Natasha Howard

**Affiliations:** ^1^Saw Swee Hock School of Public Health, National University of Singapore and National University Health System, Singapore, Singapore.; ^2^Department of Global Health and Development, London School of Hygiene and Tropical Medicine, London, UK.

**Keywords:** Healthy Societies, Health Policy, Health Systems, Healthcare Reform

## Abstract

The vision to create "Healthy Societies" is a reiteration of "Health for All" first made in the Declaration of Alma-Ata almost half a century ago. We contend that this long journey is due to "Socio-Political Inertia" that has prevented societies from transforming even in the presence of enabling policies. The analysis of policy documents by Nambiar et al could help set the stage for understanding how best to advance healthy societies, but the aspirations expressed in documents require active engagement and implementation to enable societal change. We first draw inspiration from the convergence of multiple streams in Kingdon’s model in exploring how to chart the journey toward healthy societies. We then argue that the vision of healthy societies should be articulated in ways that speak to the different societies that will own it and build coalitions to turn this vision into reality.

## Introduction

 There is no shortage of vision statements in global health. “Healthy Societies” is one of them and is the latest reiteration of the clarion call for “Health for All” first expressed in the Declaration of Alma Ata in 1978. Earlier reiterations include primary healthcare in the 2018 Declaration of Astana and the repeated call for “Health for All” in the 1986 Ottawa Charter for Health Promotion. The final 2008 report on “Social Determinants of Health” highlighted the social and economic conditions that drive health inequities. In 2015, United Nations member states adopted the Agenda 2030 Sustainable Development Goals (SDGs), including “ensuring healthy lives and promoting well-being *for all at all ages.*” “Universal Health Coverage” (UHC), championed by the World Health Organization (WHO) and governments around the world through SDG Target 3.8, is yet another reiteration of health for all. In this journal, Buse et al explained that “Healthy Societies” builds on more than 45 years of seminal initiatives since Alma Ata. They framed healthy societies as driven by societal “values” of equity, collaboration and empowerment, focused on “people” and their role in flourishing societies, linked to “place” as the setting of everyday life, impacted by “products” that either support or hinder healthy lifestyles, and are inseparable from “planet” that includes not only the natural environment but also how human progress is measured.^1^ Therefore, “Healthy Societies” reinforces a similar vision of creating a healthier world, drawing on these previous seminal initiatives and highlighting the expansion of the goal beyond the remit of government and the health sector by expressing the vision as a societal aspiration. We have been calling for a healthy world for such a long time, so why are societies still unhealthy?

 The review by Nambiar et al,^2^ which accompanied the article by Buse et al, raised this critical question of the “how” of healthy societies. With the primary intent “to guide action and political engagement for reform,” Nambiar et al analyzed 68 purposively selected documents thematically for “concepts seeking to arrive at healthy societies.” They identified three policy levers: (*a*) “regulatory and fiscal measures” enforced by an interventionist and redistributive state to address social and health inequities; (*b*) “intersectoral action” for purposeful and coordinated engagement beyond the health sector; and (*c*) “redefining measures of progress” to shift away from conventional measures (eg, gross domestic product) towards alternatives that consider societal well-being and sustainable development. In terms of the enablers that could bring these policy levers to bear, they identified (*a*) “political will and accountability,” (*b*) “social mobilization and community action,” and (*c*) “generation and use of knowledge.” However, they also underscored the lack of political solutions in mostly technical documents, the neglect of issues of power and rights-based approaches, and weak accountability.

## Healthy Societies: From Aspiration to Practice

 Nambiar and colleagues’ analysis helps mainstream the vision of healthy societies in the literature and opens space for discussion, or perhaps debate, about what is required to turn healthy societies from aspiration to practice. The authors acknowledged that their focus on policy levers and enablers excluded analysis of the barriers to policy action, which they noted as a limitation. Additionally, there is a limitation in relying on policy documents to interrogate and understand not only “what” healthy societies mean but more so “how” to achieve them. Indeed, policy statements, political declarations, reports, peer-reviewed articles, and guidance published since 1974—sources for their findings—are important because these documents could facilitate societal change. However, without effective implementation, the existence of policies and guidance alone will not create healthy societies. Several attempts at initiating societal reform across a range of settings have led to suboptimal results despite the existence of supporting policies.

 To cite an example, the Philippines passed the 2019 “UHC Act,”^3^ which many considered landmark legislation that would set in motion a package of sectoral and institutional reforms based on an explicit legal declaration that all Filipinos have a right to health. The rules and regulations to implement this law contained a comprehensive list of reforms, including expansion of the social health insurance program (ie, PhilHealth). However, six years after the law’s enactment, health sector advocates and stakeholders in the Philippines are clashing with the current government for its diversion of PHP89 billion (US$ 1.6 billion) of funds allocated for PhilHealth back to the national treasury.^4^ Concerned citizens filed a lawsuit against the government before the Supreme Court, which will adjudicate the legality of the fund transfer. Amidst this turbulence, out-of-pocket expenditure (as % of current health expenditure) in the Philippines stalled at 44.4% in 2023 (from 44.6% in 2021) despite the passage of the 2019 UHC law. In contrast, 2021 out-of-pocket expenditures of regional neighbors were much lower (eg, Malaysia 32.1%, Indonesia 27.5%, and Thailand 9.0%).^5^

 Consider Kenya and India as a second example. Both countries acceded to the Convention on the Elimination of All Forms of Discrimination Against Women (CEDAW), the principal international treaty to promote women’s rights, in 1984 and 1993, respectively. Achieving health equity and gender equality is a prerequisite for healthy societies and will dismantle gender-based discrimination and exclusion that will improve health outcomes.^6^ Over time, Kenya and India introduced legal measures to advance gender equality, including a quota reserving at least 1/3 of seats in political leadership and government service for women. However, analysis of legislation in both countries revealed several unaddressed concerns, including equal pay in India, reproductive rights in Kenya, and family life and work balance in both countries despite their ratification of CEDAW.^7^

 A third example is the SDGs themselves, much celebrated during their promulgation a decade ago as demonstrating the willingness of states—despite their differences—to rally behind a shared vision. Today’s world appears quite different, more fragmented than ever, and beset by polycrisis, including an imminent climate catastrophe. The latest SDG progress report shows, alarmingly, that only 17% of SDG targets are on track as of 2024,^8^ and no hard mechanisms exist to hold member states accountable for their failures to deliver on SDG commitments.

 These examples serve as cautionary tales that the movement to build healthy societies could suffer the same fate, ie, lofty in ambition but sluggish in execution. An apt Filipino expression, *ningas kugon*(literally, “grass fire”), describes this—referring to something that begins with great enthusiasm only to fizzle out soon after, like grass that ignites but quickly burns out.

## Moving Socio-Political Inertia Through Multiple Streams

 Nambiar et al are correct in calling attention to the importance of political solutions to complement technical initiatives and the need to address power imbalances, neglect of human rights, and weak accountability. While we acknowledge the importance of these issues, one critical need is to better understand the mechanisms for change that will address these issues and ignite action against socio-political inertia. We use “socio-political inertia” to mean the resistance of social and political systems to change despite the potential benefits and existence of enabling policies, rhetoric, and even infrastructure.

 In describing policy levers and their enablers, Nambiar et aldrew inspiration from Gaventa’s “power cube” to identify the forms, levels, and spaces of power and their interactions, or, in the authors’adaptation, the levers and enablers that could be deployed to advance healthy societies. We could also draw inspiration from Kingdon’s multiple streams model^9^ in charting the journey towards healthy societies. Developed in 1984 to explain the policy development process and widely used in the literature and policy discourse, Kingdon’s multiple streams model posits that windows of opportunity open when the three process streams of “problems, policies, and politics” converge.

 In this regard, Nambiar and colleagues’ focus on policy documents and their call for more political engagement touched on the streams of policy and politics. Perhaps we also need to further consider the problem stream—which is almost always assumed to be self-evident but may, in fact, need some reinforcement. To confront socio-political inertia, we need to “problematize” the issues that warrant the creation of healthy societies by challenging common policy assumptions and inertial interests, identifying power and unintended consequences, and (re)conceptualizing what sustainable success looks like. Those of us in public health academia, health policy, and healthcare practice well understand the problems that healthy societies seek to address. These include poor health, inequities, stagnant or worsening well-being, and insufficient resourcing and prioritization to enable sustainability. However, whether there is a wider appreciation of these problems across sectors and outside our already-fragmented global public health space is unclear.

 Five years ago, the COVID-19 pandemic opened wide the problem stream and compelled societies to problematize the pandemic’s threat to health as a crucial global concern.^10^ The rapidly evolving pandemic forced swift and agile policy decision-making,^11^ in a departure from “business as usual,” to respond to constituents who became more demanding of their right to health. The COVID-19 pandemic showed that socio-political inertia was not insurmountable and societies could transform amidst disruption. But not all crises lead to lasting societal transformation,^12^ and any windows of opportunity opened by the convergence of Kingdon’s streams can provide space for action but not necessarily determine that this action will be positive. For example, the pandemic also widened health inequities^13^ in the United States and other countries, intensifying polarization and pushing some to revert to isolationist and unilateral policy approaches, as exemplified by the inequitable access to COVID-19 vaccines between and within countries. The natural ‘wax and wane’ cycle of crises means that in the geopolitics of today, health has once again been subordinated to governments’ economic and defense priorities. Current political events, including in the United States, are undermining WHO, the only truly global health organization that can bring together states and non-state actors to make the world a healthier place.

## Staying the Course

 The world need not wait for the next major crisis to retrigger our recognition of the importance of healthy societies. Nambiar and colleagues’ work has shown existing policy documents to build upon. We need to problematize these issues, do the hard work of socio-political change, and leverage the collective power of multisectoral coalitions to advance healthy societies. Constant vigilance and activism are needed to use these “windows,” opened by the convergence of problems, policies, and politics streams, to shape healthy societies. To flesh out the analysis of concepts articulated in policy documentation, advocates can identify illustrative examples of how countries are experimenting with the multiple paths toward healthy societies. We could learn from Australia, which problematized the harmful effects of social media on the mental health and well-being of children and, through robust political engagement, introduced the first policy in the world to ban social media for children under 16.^14^ We also need to make the healthy societies vision relatable across the world to increase the likelihood of local success. Other regions can learn a lot from Asia. In the Philippines, an archipelago of 115 million people, the Department of Health promoted the vision of “Healthy *Pilipinas,*” encouraging Filipinos to adopt healthy habits. The city-state of Singapore, well-known for its emphasis on social cohesion, calls its vision “Healthier SG,” helping Singaporeans to lead healthy lifestyles. In China, a country of 1.4 billion people and the world’s second-largest economy, the government articulated its vision as “*Jiànkāng Zhōngguó* 2030” (Mandarin for “Healthy China 2030”) and made “public health a precondition for all future economic and social development.”^15^ By declaring public health the precondition for development, China’s vision exemplifies Nambiar and colleagues’ exhortation to redefine how we view societal progress. The road from Alma-Ata to healthy societies has been long and winding, but to end on a hopeful note—we have the tools and capacities to shift the socio-political inertia into a momentum for change.

## Acknowledgments

 We would like to thank Dr. AliAkbar Haghdoost, editor-in-chief, and the *International Journal of Health Policy and Management* for inviting and supporting this commentary.

## Ethical issues

 Not applicable.

## Conflicts of interest

 Authors declare that they have no conflicts of interest.
